# Peripheral Blood Brain-Derived Neurotrophic Factor as a Biomarker of Alzheimer’s Disease: Are There Methodological Biases?

**DOI:** 10.1007/s12035-017-0866-y

**Published:** 2018-01-13

**Authors:** Marta Balietti, Cinzia Giuli, Fiorenzo Conti

**Affiliations:** 1 Center for Neurobiology of Aging, INRCA, Via Birarelli 8, 60121 Ancona, Italy; 2 Geriatrics Operative Unit, INRCA, Fermo, 63023 Italy; 3 Department of Experimental and Clinical Medicine, Section of Neuroscience and Cell Biology, Università Politecnica delle Marche, Ancona, 60126 Italy

**Keywords:** Brain-derived neurotrophic factor, Alzheimer’s disease, Blood, Biomarker, Confounding variables

## Abstract

Mounting evidence that alterations in brain-derived neurotrophic factor (BDNF) levels and signaling may be involved in the etiopathogenesis of Alzheimer’s disease (AD) has suggested that its blood levels could be used as a biomarker of the disease. However, higher, lower, or unchanged circulating BDNF levels have all been described in AD patients compared to healthy controls. Although the reasons for such different findings are unclear, methodological issues are likely to be involved. The heterogeneity of participant recruitment criteria and the lack of control of variables that influence circulating BDNF levels regardless of dementia (depressive symptoms, medications, lifestyle, lack of overlap between serum and plasma, and experimental aspects) are likely to bias result and prevent study comparability. The present work reviews a broad panel of factors, whose close control could help reduce the inconsistency of study findings, and offers practical advice on their management. Research directed at elucidating the weight of each of these variables and at standardizing analytical methodologies is urgently needed.

## Introduction

**Fig. 1:**
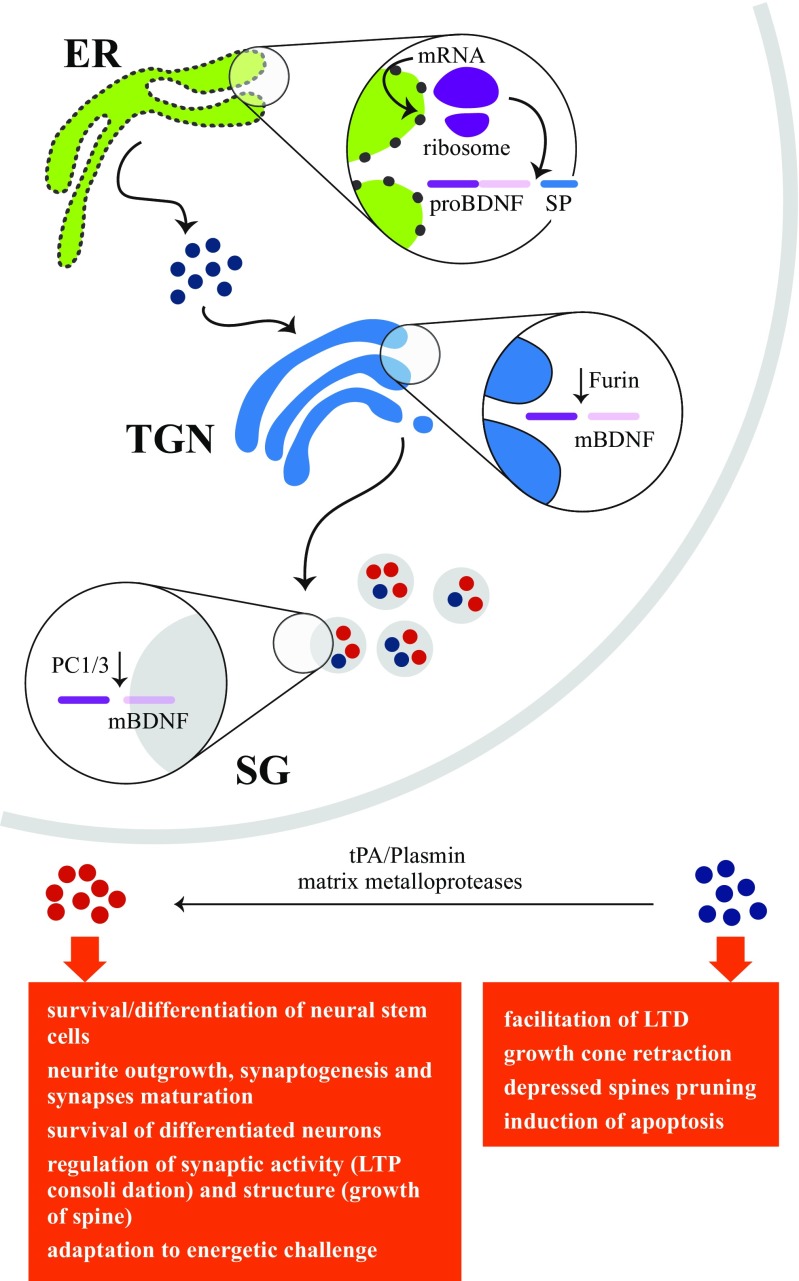
Sketch of brain-derived neurotrophic factor (BDNF) functions. BDNF, nerve growth factor, neurotrophin-3, and neurotrophin-4/5 belong to the neurotrophin family of closely related proteins. BDNF is synthesized as pre-proBDNF and converted to proBDNF by removal of the signal peptide in the endoplasmic reticulum (ER); proBDNF is then transformed to mature BDNF (mBDNF) by intracellular (in trans-Golgi network (TGN) and secretory granules (SG)) and extracellular cleavage events. Pro and mature BDNF are both active molecules playing different key roles: proBDNF through the p75^NTR^/sortilin complex and mBDNF through its receptor TrkB. Blue circles, proBDNF; red circles, mBDNF; LTD, long-term depression; LTP, long-term potentiation; tPA, tissue plasminogen activator

Brain-derived neurotrophic factor (BDNF) is a neurotrophin family member that plays key physiological functions in both the peripheral and central nervous system (CNS) (Fig. 1) [1–3]. Growing evidence that changes in cerebral BDNF levels and in the BDNF-TrkB signaling pathway may be involved in the etiopathogenesis of Alzheimer’s disease (AD) [4] has suggested that blood BDNF could be used as a biomarker for AD diagnosis, prognosis, and treatment monitoring.

Circulating BDNF derives from both peripheral [5–7] and cerebral sources, since the blood brain barrier is permeable in both directions [8]. A correlation of circulating BDNF with brain levels as well as brain phenomena has been suggested by animal and human studies [9–15]. However, investigations of its value as a biomarker of AD have given inconclusive results, since higher [16, 17], lower [18–28], or similar levels of circulating BDNF [29–33] have all been described in AD patients compared to healthy controls. Although the reasons for the conflicting reports remain unclear, a recent meta-analysis has found that nonhomogenous recruitment criteria of demented subjects and healthy controls may induce a significant bias [34].

This work delves into the issue by reviewing a broad panel of factors that can influence circulating BDNF, compromising data comparability, and offers some practical advice on how to maximize their control.

## AD Staging

Laske and colleagues [35] were the first to hypothesize that serum BDNF levels could be related to AD stage, i.e., that their early increase and subsequent reduction could depend on disease progression. Their initial upregulation has been hypothesized to be a compensatory mechanism, directed at counteracting β-amyloid accumulation, providing trophic support to offset the neuronal loss and/or promoting TAU dephosphorylation [36–41], or else a reflection of the increased choline acetyltransferase activity which characterizes the stage preceding neurodegeneration [42]. The view that different BDNF levels are found in different AD stages is shared by some researchers [43–45] and rejected by others [16, 17, 30]. Such dissimilar opinions are likely to depend on a number of factors that are discussed below. However, the inhomogeneous use of rating tools may also provide a contribution.

The Clinical Dementia Rating (CDR) scale and the Mini Mental State Examination (MMSE) are widely used tools, although the CDR scale is considered as the more reliable tool to stage AD patients [46]. The scale, which has been validated in 14 languages, assesses cognitive function using a semistructured interview for both patient and informant [47]. It evaluates a large number of domains including memory, orientation, judgment, problem solving, community affairs, household activities, hobbies, and personal care and assigns global scores that correspond to discrete levels of cognitive impairment [48]. Stringent application of the CDR would enhance comparability across studies. However, ad hoc investigations to clarify the link between AD progression and BDNF level are clearly required. Notably, patient enrolment based on strict staging criteria, i.e., mild, moderate, and severe AD (CDR 1, 2, and 3) or early, middle, and late AD (MMSE score ≥ 20, 20–10, and < 10), would ensure the recruitment of patients with similar disease stage and BDNF level, providing more homogeneous cohorts and more reliable data.

## Demographic Characteristics of Healthy and AD Cohorts

An age-related reduction in circulating BDNF has been reported both in healthy individuals and in subjects with dementia [27, 45, 49–51]. Differences have also been described between women and men [33, 49, 51–54], as has a sexual dimorphic effect of the Val66Met polymorphism on susceptibility to AD [55]. Yet, in several studies, healthy subjects and demented patients are recruited without considering these differences, and the possible influence of age and/or gender composition on cohort homogeneity is not always considered [16, 20, 27, 29]. If age and gender are used as covariates in statistical analysis, they can affect the significance of BDNF comparisons [43]. As a general rule, the presence of confounding variables, i.e., variables that can influence the outcome of interest irrespective of the study design, should always be verified before performing the analysis [56], and dedicated statistical methods employed like, for instance, model adjustment for those variables [57].

## Depressive Symptoms

Several lines of evidence indicate that cerebral BDNF is an effector of and a therapeutic target for depression [58]. BDNF signaling is significantly reduced in the hippocampus and prefrontal cortex of depressed subjects [59], and the ability of antidepressants and electroconvulsive shock treatment to induce neural plasticity appears to be closely related to BDNF signaling stimulation in these circuits [60]. Notably, the role of cerebral BDNF is area-specific, since an increase in BDNF levels in the brain reward system produces depressive symptoms [61]. With regard to the peripheral consequences of depression, a reduction in serum and plasma BDNF has repeatedly been reported [62, 63], even though its functional role is unclear. A reduction in circulating BDNF may reflect changes at the level of the CNS and/or depend on defective platelet release [64]. Since up to 50% of AD patients suffer from depression, making this disorder one of the most frequent comorbidities in such patients [65], it would be critical to discriminate depression-related from dementia-related BDNF changes.

This could be achieved in two steps. The first would involve excluding from AD cohorts those patients who suffer from clinical depression [16, 19, 20, 32, 33, 35, 43, 66] or by assessing its influence on BDNF levels [21, 45]. Indeed, whereas Lee and colleagues [21] have found no difference in serum BDNF between AD patients with and without severe depression, in another study [33], apathy associated to mild dysphoria symptoms, suggestive of subclinical depression, determined a significant reduction in serum BDNF in affected versus non-affected AD subjects. Furthermore, the difference in BDNF concentrations in platelet-rich plasma between AD patients and healthy subjects, found by Platenik and colleagues [45], has been confirmed in patients with depression, but not in those without it. Again, recruitment criteria play a critical role.

The second step would be the evaluation of depressive mood in healthy and AD cohorts. Even though this element may be a confounding variable, the few published data are not sufficient to draw conclusions, because they have been obtained with different methodological approaches. Most studies have used the Geriatric Depression Scale (GDS) [27, 31, 43, 45], but other diagnostic protocols have also been applied [16]. Moreover, whereas AD and healthy subjects have mostly been kept separate [16, 43, 45], in some studies they have been pooled [27, 31]. Here, too, uniformity is vital, especially where the protocol of depressive mood evaluation is concerned. Despite the existence of several depression rating scales [67], the GDS has several advantages. In fact, it was specifically designed to assess mood status and identify depressive symptoms in the elderly; moreover, it focuses on psychological aspects, avoiding symptoms that may overlap with medical disorders or aging. Although it had originally been based on 30 items, a 15-item version has subsequently been developed [68] and is the one used in most validation studies.

## Medications

Several psychoactive drugs administered to AD patients influence circulating BDNF. Acetylcholinesterase inhibitors [20] and antidepressants [69] increase peripheral BDNF, whereas benzodiazepines reduce it [24, 70], even though it is unclear whether the changes are related to cerebral events or are rather epiphenomena. Despite their key importance, the effects of medications have been ignored in numerous studies [18, 19, 21–25, 27–30, 32, 35, 45]. The works that have considered them have adopted one of two approaches: (i) enrolment of naïve subjects or of patients in the washout phase [20, 33, 66] or (ii) evaluation of the consequences of medication use [16, 17, 24, 31, 43]. Although both approaches provide useful data, the latter sketches a clearer picture of the average condition of the population and could therefore be more useful to test the biomarker function of circulating BDNF in clinical practice. Besides psychoactive drugs, lipid-lowering drugs [51, 71] and antidiabetics [72] are also widely prescribed to the elderly and affect BDNF levels. These drugs should therefore be factored in, as should all medications influencing platelet activity, like nonsteroidal anti-inflammatories, anticoagulants, and antihypertensives (see section “Lack of Overlap Between Serum and Plasma BDNF”). Only a limited number of investigations have assessed psychoactive as well as nonpsychoactive medications [16, 20, 33, 43]. Moreover, some studies have examined medication use in AD as well as healthy elderly subjects, recognizing the fact that cognitively healthy elderly subjects are also often treated for hypertension, diabetes, coagulation defects, or dyslipidemia [16, 17, 33, 43, 66]. Clearly, all potentially interfering medications should be considered in patients and controls alike, for instance by a dichotomous analysis where the BDNF levels of medication users and nonusers in both groups are kept separate and/or combined.

## Lifestyle

Daily habits like exercise, smoking, alcohol consumption, and eating behavior can influence circulating BDNF levels; yet, they are rarely and not exhaustively analyzed [16, 25, 32, 43, 45, 66].

Exercise, especially aerobic activity, is known to increase circulating BDNF [51, 73, 74], mainly through stimulation of its production in the brain [75–78], although release from peripheral sources is probably also stimulated [79, 80]. Yet, an inverse relationship with peripheral BDNF has been found when patients enrolled in studies have been examined for daily physical activity (not training programs) [81]. Although the reason for the discrepancy is unclear, improved cardiorespiratory fitness [82, 83] and energy metabolism [84] in spontaneously active persons may play a key role. It is reasonable to consider that healthy elderly subjects have different rates of physical activity compared to AD patients [25, 85] and that this influences BDNF levels irrespective of dementia. A simple and effective tool to assess daily physical activity in the aged is the Physical Activity Scale for the Elderly (PASE) [86]. The scale has been designed for epidemiological studies to rate leisure time, household work, and job tasks in individuals aged 65 years or older. Therefore, the PASE score of healthy and AD cohorts should always be compared and considered as a possible covariate.

The limited available evidence regarding the effect of smoking on circulating BDNF points at a different influence on its plasma and serum levels, which are respectively lower [87, 88] and higher [51, 89–91] in smokers than in nonsmokers. A single study has found opposite results, which the authors have been unable to explain [92]. A direct influence of nicotine on cerebral BDNF has been described. Chronic nicotine administration upregulates BDNF in the cortex and hippocampus [93, 94] and in dopaminergic brain areas including the nucleus accumbens, ventral tegmental area, and substantia nigra [95, 96], but it downregulates neurotrophin in the striatum [97]. Differences in the effect of nicotine are related to exposure duration—short-term administration reducing and long-term administration increasing hippocampal BDNF [98]—and to the amount of nicotine consumed, since high but not low chronic doses reduce BDNF in the dorsal striatum [99]. The modulation of peripheral BDNF by smoking may reflect cerebral phenomena, but influences from peripheral sources may be also crucial. Indeed, in vitro analyses have documented that cigarette smoke extracts induce a dose-dependent release of BDNF, indicating that abnormal BDNF trafficking from smokers’ platelets can contribute to changes in circulating BDNF [92]. Although an in-depth analysis of the functional relationship between smoking and BDNF is beyond the scope of this paper, the above data clearly show the vital importance of classifying subjects, both healthy individuals and AD patients, as current, former, and nonsmokers. In addition, given that a significant, positive correlation has been found among serum BDNF, years of smoking [89], and smoking intensity [51, 100, 101], smokers should also be evaluated for the number of cigarettes smoked and the age when the habit was acquired. Notably, since smoking cessation induces a significant increase in plasma BDNF [87, 88], a sufficient washout period enabling normalization should be considered when assessing former smokers: to date, they have only been studied at 12 weeks, and even though BDNF had already begun to decline after 4 weeks, at 3 months, it had not reached a steady state [87]. Considering the abstinence period as a confounding factor could be an acceptable compromise.

BDNF plays an acknowledged role in addiction [102]. Accordingly, the report of a functional relationship between cerebral BDNF and alcohol use is not surprising. Moderate alcohol consumption seems to upregulate BDNF—triggering a homeostatic mechanism aimed at suppressing further intake—whereas altered BDNF signaling due to chronic alcohol use may contribute to abuse behaviors [103]. Inconsistent data are available regarding the influence of alcohol on circulating BDNF. Compared to healthy controls, subjects with alcohol dependence have been found to have unchanged [104–106] or decreased [107] serum BDNF and unchanged [107], higher [108], or lower [109] plasma BDNF. Conflicting results have also been described on the effect of abstinence from alcohol, since withdrawal has been reported both to exert no influence on plasma [110, 111] and serum BDNF [105] or to increase its serum level [104, 106, 110]. Again, the factors that can be invoked to account for these discrepancies include the heterogeneity of patients’ clinical characteristics [107]; a familial predisposition to dependence [109], a widely underrated factor; and the lack of a standardized medication schedule during withdrawal [105]. Notably, the studies that have examined the effect of alcohol on circulating BDNF have largely considered male individuals and abuse behavior, whereas information on females and moderate, social drinking is scanty. Nonetheless, some basic rules to guide the clinician in sample assessment are easy to identify and apply: healthy subjects and AD patients should all be screened for alcohol abuse and addiction and these should be considered as exclusion criteria.

Several lines of evidence support the contribution of BDNF to the dys/regulation of eating behavior: BDNF and its receptor TrkB are localized in hypothalamic and hindbrain nuclei involved in energy homeostasis [112, 113], associations have been found between BDNF polymorphisms (Val66Met, -270C/T, 196G/A) and anorexia and bulimia nervosa [114–116], and animal models of BDNF alterations exhibit hyperphagia and obesity [117, 118]. Eating disorders affect circulating BDNF, but whereas patients with anorexia and bulimia nervosa show consistently lower serum BDNF [119–121], data from obese subjects are contrasting, since higher [119, 122–124], lower [125], or unchanged serum levels [126], and lower [127, 128] or unchanged levels of plasma BDNF [127, 129, 130] have been described in obese compared to normal-weight subjects. These inconclusive findings may partly be explained by the fact that obesity is a chronic, inflammatory condition with negative systemic effects that may include effects on platelet storage [122] and BDNF production by peripheral blood mononuclear cells [130], with consequences that may vary in relation to disease severity, comorbidities, and medications. The report by Heun and colleagues [131] that eating disorders are more frequent among AD patients than among healthy elderly controls suggests that severely under- and overweight subjects should not be enrolled. Another useful option would be to assess circulating BDNF in healthy and AD subjects divided according to the WHO body mass index (BMI) classes defined for the adult population (i.e., moderate/severe thinness, BMI ≤ 17 kg/m^2^; underweight, 17 < BMI < 18.5 kg/m^2^; normal weight, 18.5 ≤ BMI < 25.0 kg/m^2^; overweight, 25.0 ≤ BMI < 30.0 kg/m^2^; obesity, BMI ≥ 30.0 kg/m^2^; obesity class 3, BMI > 40.0 kg/m^2^) [132]. Interestingly, a correlation between BMI and circulating BDNF (positive for serum and negative for plasma) has been found in patients with eating and metabolic disorders [49, 121, 123, 124, 127], but little or no information is available for patients with dementia. The single study assessing the possible influence of BMI on plasma and serum BDNF in AD patients has found no effect [45]. The issue clearly deserves further analysis.

## Lack of Overlap Between Serum and Plasma BDNF Levels

The average serum BDNF level is about 200-fold higher that of plasma, a difference that reflects the amount of BDNF that is stored in circulating platelets and released during clotting [5].

Platelets undergo extensive changes in AD, including hyperactivation [133], impairment of oxidative state [134], mitochondrial deficiencies [135], basal changes in membrane fluidity and cholesterol levels [136], and atypical amyloid precursor protein (APP) metabolism [137]. Some platelet dysfunctions, such as an elevated percentage of the coated subpopulation [138], changes in APP isoform ratio [139], and increased basal activation [140], have been related to AD progression. By influencing thrombocyte function, these phenomena may exert different and even opposite effects on serum and plasma BDNF; as a consequence, different results may be obtained from the two matrices [141]. Despite its potential importance, only two studies have analyzed the effect of platelet changes on plasma and serum BDNF in AD. Platenik and colleagues [45] showed that the reduced BDNF levels found in platelet-rich plasma in patients with moderate and severe AD were due to a reduced platelet number, not to reduced BDNF levels in thrombocytes, whereas Laske et al. [66] reported that serum BDNF levels in AD patients significantly correlated with the level of plasma β-thromboglobulin, a marker of platelet activation that is unrelated to plasma BDNF.

Additional factors besides dementia can differentially affect plasma and serum BDNF in AD patients, including proinflammatory cytokines [142] and medications [143–145]. The importance of caution when extending any conclusion has been confirmed by a recent meta-analysis [146] reporting reduced serum but unchanged plasma BDNF in AD compared to healthy subjects. This work, albeit informative, did not however consider key confounding factors such as dementia staging and lifestyle and assessed the possible influence of medications only in patients, not in healthy controls. Until fresh experimental data establish which peripheral matrix is the better mirror of cerebral BDNF, simultaneous evaluation of plasma and serum levels seems to be the safest approach, not only because it may offset the influence of platelet function but also because plasma and serum may provide different types of information. Indeed, whereas plasma BDNF is the “active” form of the molecule, i.e., the fraction that is available for crossing the blood brain barrier through a saturable transport system, serum BDNF, which reflects platelet amount, may rather provide a long-term marker [15, 54, 144].

## Experimental Issues

To complete the analysis, here are some considerations on a number of methodological features that, albeit easier to control than the varied factors described above, are however underrated.

Two preanalytical conditions can be critical.

(i) BDNF has been suggested to follow a circadian rhythm in humans, peak values being reached in the morning [147, 148]. Differences between plasma and serum and between genders have also been documented, with serum levels being more stable throughout the day in females [149]. In all studies, the subjects tested for daily variations were young or adult, involving that further work is needed to measure these data in the elderly. In all cases, the time of blood sampling should be consistent and carefully enforced.
(ii) Little or no information tends to be reported about the duration of the sample storage period, even though Trajkovska and colleagues [150] reported that while BDNF levels in whole blood remain stable for up to 5 years at − 20 °C, serum levels are negatively affected by protracted storage. A significant correlation between lower serum BDNF and longer storage time has been confirmed by Bus et al. [89], who also demonstrated that a temperature of − 85 °C attenuates the phenomenon. Although immediate assays are likely to remove any possible bias, a limited, uniform storage time (i.e., a few months) should always be applied.

Materials and methods to detect the biomarker are also crucial. Circulating BDNF is easily measured using commercial ELISA kits that seem the best approach considering future clinical applications. Numerous kits are available, and comparing five of them, Polacchini et al. [151] evidenced that differences exist in their performances, especially regarding interassay variation and specificity for total rather than mature BDNF. This finding stressed that the definition/validation of adequate standards of reliability is extremely urgent. Unfortunately, up to date, we cannot suggest the best company(ies) as the analysis of the complete panel of all existing kits is lacking. An effort to fill this gap should be a priority with the involvement of different laboratories in a systemic program.

As noted above, statistical analysis may be critical. The distribution of the variable “blood BDNF” is not suitably considered. A nonnormal distribution has widely been reported [17, 43, 45, 50, 54, 105, 108, 119, 150], albeit with some exceptions [33, 52, 87]. The phenomenon may go undetected in small samples. Moreover, the assessment of variable distribution is not explicitly described in several studies, thus raising doubts on the use of parametric tests. Normality should always be verified, and if distribution is not normal, nonparametric statistics [152] or variable transformation [153] should be applied.

## Conclusions

**Fig. 2:**
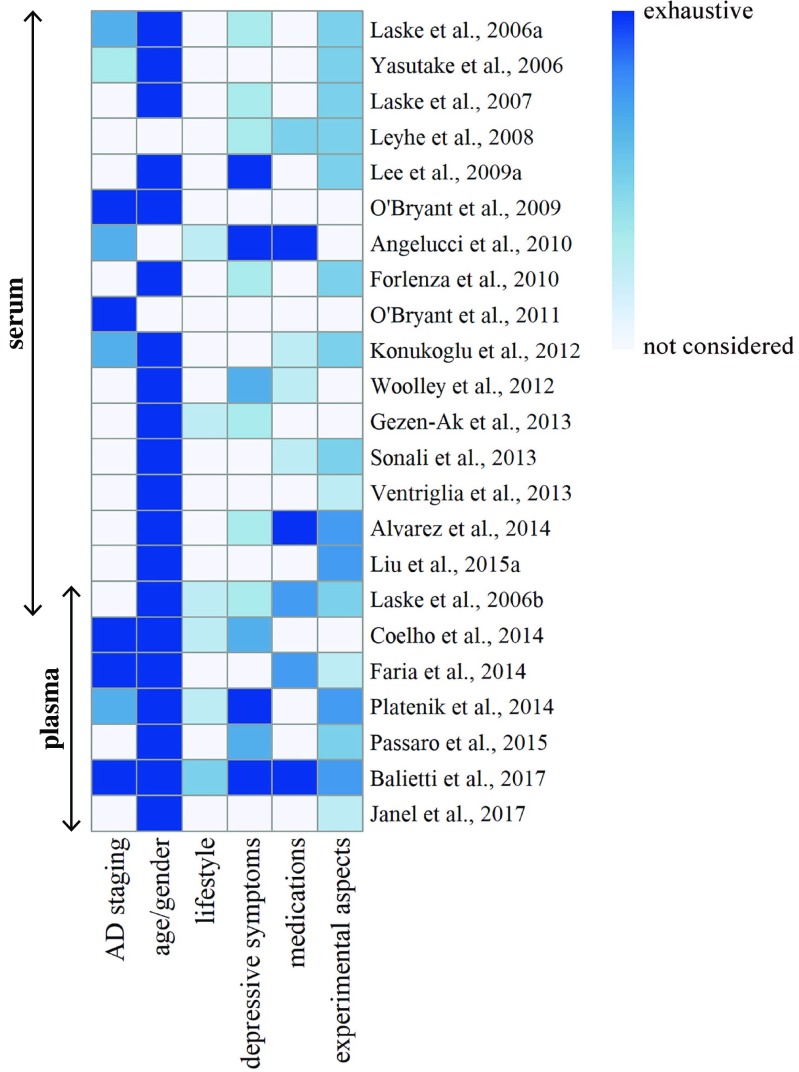
Representation of the degree of control exerted on the various factors. The heatmap outlines the level of control (indicated by the values of colors) that is exerted on each factor that may introduce a bias in the studies that have tested blood BDNF as a possible biomarker of AD. Colors range from noncontrolled (white) to completely controlled (dark blue) on the basis of the following categories: *AD staging*, (1) not considered; (2) defined by FAS (Functional Assessment Staging) score; (3) defined by MMSE (Mini Mental Stage Examination) score; (4) defined by CDR (Clinical Dementia Rating) score; *age/gender*, (1) differences between healthy and AD cohorts not adjusted; (2) healthy and AD cohorts without differences or with differences between healthy and AD cohorts adjusted; *lifestyle*, (1) not evaluated; (2) evaluation of one factor; (3) evaluation of two factors; (4) evaluation of three factors; (5) evaluation of four factors; *depressive symptoms*, (1) not evaluated; (2) exclusion of patients with a clinical diagnosis of depression or assessment of their weight; (3) evaluation of depressive symptoms; (4) exclusion of patients with a clinical diagnosis of depression or assessment of their weight and evaluation of depressive symptoms; *medications*, (1) not evaluated; (2) evaluation of psychoactive medications in AD patients; (3) evaluation of psychoactive and nonpsychoactive medications in AD patients; (4) evaluation of psychoactive medications in AD patients and healthy subjects; (5) evaluation of psychoactive and nonpsychoactive medications in AD patients and healthy subjects; *experimental aspects*, (1) not evaluated; (2) evaluation of data normality; (3) control of time of blood sampling; (4) evaluation of data normality and control of time of blood sampling; (5) evaluation of data normality, control of time of blood sampling, and information about storage duration

Figure 2 schematizes the degree of control (noncontrolled, partially controlled, completely controlled) that has been exerted on each potential confounding factor in the studies that have tested blood BDNF as a possible biomarker of AD. Lifestyle is by far the most underestimated area, since none of the studies have considered all four components (exercise and smoking, drinking and eating habits), and none of the components have been analyzed in depth. AD staging is also a thorny issue, since in more than half of the studies the patients’ cohort was not rigorously classified and the remaining investigations did not adopt homogenous criteria. The assessment of the influence of medications is insufficient, considering the limited attention given to polypharmacy in nondemented elderly people and to nonpsychoactive molecules in healthy and AD subjects. In contrast, there seems to be a greater awareness of the critical importance of depression and of the need for balancing age and gender in healthy and AD cohorts.

Where the experimental issues are concerned, the total lack of information about storage duration is maybe the most stunning aspect, even though this does not necessarily imply a real lack of standardization. Finally, the fact that a single study tested serum as well as plasma stresses the need for comparing the two matrices in the same cohorts, to clarify their different, possibly even complementary, nature.

The main conclusion that can be drawn from this review is that result comparability and extension are critically impaired by the lack of control on factors that can influence circulating BDNF irrespective of dementia. To shed light on the actual value and reliability of circulating BDNF as a biomarker of a complex condition such as AD, further work devoted to elucidating the weight of each factor and at standardizing methodologies is urgently required.
